# Conserved Noncoding Elements Influence the Transposable Element Landscape in *Drosophila*

**DOI:** 10.1093/gbe/evy104

**Published:** 2018-05-29

**Authors:** Manee M Manee, John Jackson, Casey M Bergman

**Affiliations:** 1 Faculty of Life Sciences, University of Manchester, Manchester, United Kingdom; 2 National Center for Biotechnology, King Abdulaziz City for Science and Technology, Riyadh, Saudi Arabia; 3 Center of Excellence for Genomics (CEG), King Abdulaziz City for Science and Technology, Riyadh, Saudi Arabia; 4 Department of Animal and Plant Sciences, University of Sheffield, Sheffield, United Kingdom; 5 Department of Genetics, University of Georgia, Athens, GA; 6 Institute of Bioinformatics, University of Georgia, Athens, GA

**Keywords:** noncoding DNA, conserved noncoding elements, purifying selection, transposable elements, Drosophila

## Abstract

Highly conserved noncoding elements (CNEs) constitute a significant proportion of the genomes of multicellular eukaryotes. The function of most CNEs remains elusive, but growing evidence indicates they are under some form of purifying selection. Noncoding regions in many species also harbor large numbers of transposable element (TE) insertions, which are typically lineage specific and depleted in exons because of their deleterious effects on gene function or expression. However, it is currently unknown whether the landscape of TE insertions in noncoding regions is random or influenced by purifying selection on CNEs. Here, we combine comparative and population genomic data in *Drosophila melanogaster* to show that the abundance of TE insertions in intronic and intergenic CNEs is reduced relative to random expectation, supporting the idea that selective constraints on CNEs eliminate a proportion of TE insertions in noncoding regions. However, we find no evidence for differences in the allele frequency spectra for polymorphic TE insertions in CNEs versus those in unconstrained spacer regions, suggesting that the distribution of fitness effects acting on observable TE insertions is similar across different functional compartments in noncoding DNA. Our results provide evidence that selective constraints on CNEs contribute to shaping the landscape of TE insertion in eukaryotic genomes, and provide further evidence that CNEs are indeed functionally constrained and not simply mutational cold spots.

## Introduction

Transposable elements (TEs) are mobile DNA sequences that make up a significant fraction of the genomes of many multicellular organisms (Elliott and Gregory 2015), including the model insect species, *Drosophila melanogaster* (Bergman et al. 2006; Sackton et al. 2010). TEs are powerful mutagenic agents that can affect gene expression and genome stability and are responsible for the majority of spontaneous mutations in *D. melanogaster* (Ashburner et al. 2005). While many gaps remain in our understanding of the mechanisms that control TE content in natural populations of *D. melanogaster*, it is well established that TE insertions in the *D. melanogaster* genome are largely restricted to noncoding DNA (reviewed in Barron et al. 2014). Early restriction mapping studies on a limited number of loci revealed that large DNA insertions (assumed to be TEs) were rarely found in transcribed regions (Aquadro et al. 1986, 1992; Langley and Aquadro 1987; Langley et al. 1988; Schaeffer et al. 1988). Subsequent analysis of the *D. melanogaster* reference genome showed that the paucity of TEs in transcribed regions is primarily driven by a strong depletion of the number of TE insertions in exons combined with a weaker reduction in introns (Kaminker et al. 2002; Lipatov et al. 2005). More recently, analysis of population genomic data has confirmed that TE insertions are rare in *D. melanogaster* exonic regions (Kofler et al. 2012; Cridland et al. 2013; Zhuang et al. 2014).

The underrepresentation of TEs in *D. melanogaster* exons is most likely explained by natural selection purging TE insertions that disrupt gene function from natural populations (Lipatov et al. 2005; Petrov et al. 2011; Kofler et al. 2012). In general, TE insertions in *D. melanogaster* are thought to be under some form of purifying selection, based on the observation that they typically have lower allele frequencies relative to single nucleotide polymorphisms (SNPs) from the same population (Aquadro et al. 1986, 1992; Langley and Aquadro 1987; Langley et al. 1988; Schaeffer et al. 1988; Cridland et al. 2013). However, few studies have directly investigated the allele frequency distribution of TE insertions in exons, principally because of the lack of data, and past studies have led to mixed conclusions. Analysis of a small sample of exonic TE insertions using a pool-PCR strategy suggested their allele frequencies did not differ substantially from nonexonic TE insertions with similar genomic properties (Lipatov et al. 2005). In contrast, genome-wide analysis using pool-seq data showed a reduction in median allele frequencies for TE insertions in exons relative those found in intergenic regions (Kofler et al. 2012).

In addition to effects manifest at the RNA or protein level, it is also possible TE insertions may be selected for their effects at the DNA level in noncoding regions, for example, by interfering with *cis*-regulatory elements (Geyer et al. 1990; Lerman and Feder 2005). While comprehensive *cis*-regulatory maps for *D. melanogaster* remain incomplete (Negre et al. 2011; Arnold et al. 2013), it is well established that highly conserved noncoding elements (CNEs) are an abundant component of the *D. melanogaster* genome (Bergman and Kreitman 2001; Siepel et al. 2005) and that CNEs often overlap with known *cis*-regulatory elements (Emberly et al. 2003; Brody et al. 2012). It has been estimated that 30–40% of sites in *D. melanogaster* noncoding DNA are contained in CNEs (Siepel et al. 2005), and population genetic analysis has shown that these CNEs are maintained by purifying selection (Casillas et al. 2007). Thus, CNEs represent an abundant class of noncoding features under purifying selection that may influence the landscape of TE insertions. Previous work showed that artificially induced TE insertions are depleted in the most highly conserved CNEs (so-called “ultra-conserved elements”) (Makunin et al. 2013). However the nonrandom target preferences, requirement for marker gene activation in TE detection, and experimental origin of the TEs analyzed by Makunin et al. (2013) do not allow conclusions to be drawn about CNE-based constraints on TE insertion for the endogenous set of TE families in natural populations. Resolving whether CNEs influence the landscape of TE insertion in natural populations of *D. melanogaster* will provide further insight into the factors governing TE dynamics in this species, and contribute to our broader understanding of the forces that shape genome organization and molecular evolution in general.

Here, we use genome-wide data sets of “nonreference” TE insertions (i.e., TEs identified in a resequenced sample that are not present in the reference genome) from a North American population of *D. melanogaster* (Linheiro and Bergman 2012; Mackay et al. 2012; Zhuang et al. 2014) to investigate whether selective constraints on CNEs influence the landscape of TE insertions in noncoding DNA. These data sets allow unprecedented insight into this fundamental question by providing large samples of naturally occurring TE insertions mapped at nucleotide-level resolution in individual strains of *D. melanogaster*. We initially establish that signals consistent with purifying selection can be observed in our data by confirming past results that the abundance of TE insertions is strongly reduced in exonic regions and weakly reduced in intronic regions relative to intergenic regions. We then show that the abundance of TE insertions is significantly reduced in both intronic and intergenic CNEs relative to random expectations. However, the proportion of TE insertions we estimate to be eliminated from CNEs is lower than in exonic regions, suggesting that many noncoding functional elements harbor TE insertion mutations in natural populations of *D. melanogaster*. We also find no evidence that the derived allele frequency (DAF) spectrum for TE insertions inferred from strain-specific genome sequences varies significantly across different functional compartments of the *D. melanogaster* genome. Our results are consistent with selective constraints on CNEs in noncoding regions acting to influence the landscape of TE insertion in *D. melanogaster*. However, our results also suggest that the evolutionary forces governing the abundance of TE insertions in different functional compartments of the *D. melanogaster* genome may be decoupled from those controlling the allele frequency of observable TE insertions in natural populations.

## Materials and Methods

### Data Sets

Annotations of genes (flyBaseGene), TEs in the reference genome (rmsk), and conserved elements (phastCons15way) on Release 5 (dm3) coordinates of the *D. melanogaster* genome were obtained from UCSC Genome Browser (Siepel et al. 2005; http://www.repeatmasker.org/ (last accessed June 6, 2018); Gramates et al. 2017; Tyner et al. 2017). Annotations of nonreference TE insertion in the *Drosophila* Genetic Reference Panel (DGRP) of *D. melanogaster* strains from Raleigh, NC (Mackay et al. 2012) were obtained from Supplementary Material of papers describing two different TE detection methods: ngs_te_mapper (Linheiro and Bergman 2012) and TEMP (Zhuang et al. 2014). These data sets were chosen because both ngs_te_mapper and TEMP take advantage of the TE-flanking regions information contained in split reads and thus localize TE insertions to precise genomic coordinates.

The ngs_te_mapper data set consists of nonreference TE insertions from 37 long terminal repeat (LTR) retrotransposon and terminal inverted repeat (TIR) transposon families on the major chromosome arms (chrX, chr2L, chr2R, chr3L, chr3R, and chr4) identified using split-read information in whole-genome Illumina shotgun sequence data from 166 DGRP strains (Linheiro and Bergman 2012). A new BED file for this data set was generated by Dr Linheiro R (personal communication) that encodes the number of DGRP strains in which each insertion was found in the score column (https://figshare.com/articles/Alternate_version_of_File_S4_from_Linheiro_amp_Bergman_2012/1168883; last accessed June 6, 2018). The TEMP data set consists of nonreference TE insertions from 56 LTR retrotransposon, non-LTR retrotransposon, and TIR transposon families identified using read-pair and split-read information in whole-genome Illumina shotgun sequence data for 53 DGRP strains (Zhuang et al. 2014). We transformed the original TEMP data set from https://zlab.umassmed.edu/TEMP/TEMP_resources/DGRP_53lines_TE_polymorphisms.tar.gz (last accessed June 6, 2018) to match the format of the ngs_te_mapper data set as follows. TE insertions in the original *.insertion.refined.bp.refsup TEMP output files were first merged across all strains. Insertions supported by split-read data on both ends of the TE (“1p1” flag) that are mapped to precise genomic coordinates on the major chromosome arms (chrX, chr2L, chr2R, chr3L, chr3R, and chr4) were then extracted and converted to BED format. BED-formatted insertions were then sorted and clustered using BEDtools complement (-s -d 0) (Quinlan and Hall 2010). The number of strains per cluster containing a TE insertion for the same TE family on the same strand was then encoded in the score column of a BED-formatted file. For both data sets, a small number of TE insertions were predicted to occur at the same location, either from closely related TE families (e.g., *Stalker* vs *Stalker 4*) or for TIR elements predicted on opposite strands at the same location (e.g., *S* element). We kept one of these redundant annotations based on the first occurrence in the data set. Finally, we excluded all *P* element insertions from both data sets, since this TE family is known to have a strong nonrandom preference to insert around transcriptional start sites (Spradling et al. 1995; Bellen et al. 2004; Kofler et al. 2015).

### Assigning TE Insertions to Genomic Compartments

We partitioned regions of the *D. melanogaster* genome into mutually exclusive exonic, intronic, and intergenic compartments based on the gene structures in the dm3 flyBaseGene track using the overlapSelect and BEDtools intersect, complement, and subtract tools (Quinlan and Hall 2010; Kuhn et al. 2013). Each tool was run using default parameter settings. Our partitioning strategy follows Lipatov et al. (2005) and assumes a hierarchy of functional constraints for genomic regions that have multiple annotation states due to alternative splicing or promoter usage: namely, functional constraints on exonic regions take precedence over intronic regions, and constraints on intronic regions take precedence over intergenic regions. Exonic regions span the union of all exon intervals in the genome and include both coding sequences (CDS) and untranslated regions (UTRs). Intronic regions were defined as the complement of exonic regions in genomic intervals spanned by at least one transcript model. Intergenic regions were defined as the complement of all exonic and intronic regions. Intronic and intergenic regions were further partitioned into CNEs and spacers using the dm3 phastCons15way track. Spacers are defined as the noncoding regions complementary to CNEs that exhibit low primary sequence conservation (Bergman et al. 2002; Casillas et al. 2007).

We restricted our analysis to regions of the *D. melanogaster* Release 5 genome sequence with normal rates of recombination using criteria established in previous population genomic analyses of TEs in *D. melanogaster* (Cridland et al. 2013, 2015): chrX: 300000–20800000, chr2L: 200000–20100000, chr2R: 2300000–21000000, chr3L: 100000–21900000, chr3R: 600000–27800000. Low recombination regions on the major chromosome arms (including all of chr4) were excluded because of the high density of reference TE sequences in these regions (Bartolome et al. 2002; Bergman et al. 2006), which poses challenges both to identifying nonreference TE insertions and to defining CNEs using comparative genomic data. Additionally, we excluded regions of the reference genome identified by RepeatMasker as TE from normally recombining regions because nonreference TEs are likewise systematically underpredicted in these regions. Low-recombination and reference TE intervals were subtracted from all exonic, intronic, intergenic, CNE, and spacer compartments. Normally recombining regions excluding reference TE intervals occupy 86.6% of the 120 Mb *D. melanogaster* Release 5 genome.

Nonreference TE insertions in high recombination regions excluding reference TE intervals were then assigned to genomic compartments using overlapSelect (Kuhn et al. 2013). The locations of the nonreference TE insertions in the ngs_te_mapper and TEMP data sets analyzed here are annotated as their target site duplication (TSD) (Bergman 2012; Linheiro and Bergman 2012; Zhuang et al. 2014), which span small intervals (typically <10 bp) on reference genome coordinates and can therefore overlap the boundaries of neighboring genomic compartments. To avoid counting TEs that overlap boundaries multiply or partially in different compartments, a series of filtering steps was implemented to identify TE insertions that overlap intronic/exonic, intergenic/exonic, and CNE/spacer boundaries. Each distinct category of “overlapping” TE insertions is mutually exclusive with other overlapping or “pure” compartments. TE insertions observed in low-recombination or reference TE intervals were eliminated from both data sets. The majority of nonreference TEs in both data sets studied here were located in normally recombining regions excluding reference TE intervals (ngs_te_mapper: *n* = 6,061/6,747, 89.8%; TEMP: 4,652/5,331, 87.3%). The final data sets of nonreference TE insertions in normally recombining regions excluding reference TE intervals are available in Additional Files 1 and 2, for ngs_te_mapper and TEMP, respectively.

### Testing for Purifying Selection on TE Insertions

We tested for depletion of TE insertions in different genomic compartments relative to random expectations using a permutation approach. In contrast to goodness-of-fit tests based on expected proportions of genomic compartments, our permutation approach accommodates the fact that nonreference TEs can span multiple compartments (see above) and accounts for the empirical length distributions of intervals in different genomic compartments and the variable lengths of TSDs for nonreference TEs. Random TE insertion was simulated using BEDTools shuffle to permute the location of TE insertions in different compartments of the Release 5 genome. Random TE insertions were required to be placed within their same chromosome (*-chrom* option) and were not allowed to overlap each other (*-noOverlapping* option). Random insertions were not allowed to land in low-recombination regions or regions of the reference genome annotated as TE by RepeatMasker (Smit et al. 2013) using the BEDtools shuffle *-excl* option. These constraints were implemented for several reasons. First, as noted above, nonreference TE detection systems systematically underpredict in repetitive regions like reference TEs, which are enriched in low recombination regions. Second, reference TE spans are found almost exclusively in noncoding regions (Kaminker et al. 2002; Lipatov et al. 2005) and, within noncoding regions, reference TEs are found almost exclusively in spacers since few TE insertions in the *D. melanogaster* genome occurred prior to speciation (Caspi and Pachter 2005; Bergman and Bensasson 2007; Sackton et al. 2010). If not controlled for, the combined effects of detection bias and nonrandom distribution of reference TEs would lead to an excess of nonreference insertions in regions enriched in reference TEs in permuted data sets, even under the null hypothesis of random insertion. Finally, the efficacy of natural selection on individual alleles is reduced in regions of the *Drosophila* genome with low rates of recombination because of the confounding effects of selection on linked sites extending over larger regions (Presgraves 2005; Haddrill et al. 2007). The *-seed* option was used to allow results of each run to be replicated. TE insertions in permuted data sets were then assigned to genomic compartments as described earlier.

A series of permutation tests were performed to test the null hypothesis of random TE insertion across various sets of genomic compartments. TE insertions and intervals for compartments not included in a particular test were excluded using the BEDtools shuffle *-excl* option. All permutation tests were restricted to normally recombining regions of the genome excluding reference TE intervals as described earlier. First, TE insertions observed in all compartments were allowed to randomly insert into all compartments to test if TEs are depleted in pure and overlapping exonic regions relative to noncoding DNA. This analysis was performed as a positive control to determine if our approach could replicate previously reported results. Second, TE insertions observed in noncoding regions were allowed to randomly insert in noncoding regions to test if TEs are depleted in introns relative to intergenic regions, independent of the effects of purifying selection on exonic regions. Third, TE insertions observed in intronic regions were allowed to randomly insert in intronic regions to test if TEs are depleted in intronic CNEs relative to intronic spacers, independent of the effects of purifying selection on exonic or intergenic regions but accounting for potential selection on introns. Finally, TE insertions observed in intergenic regions were allowed to randomly insert in intergenic regions to test if TEs are depleted in intergenic CNEs relative to intergenic spacers, independent of the effects of purifying selection on exonic or intronic regions. For each test, 10,000 permutations were performed to provide a distribution of outcomes under the null hypothesis of random insertion. *P* values under the null hypothesis of random insertion were estimated as the proportion of 10,000 permutations with numbers of TE insertions in putatively selected compartments that were less than or equal to the observed data. We tested the one-sided hypothesis that putatively functional categories should have a depletion of TE insertions. To conservatively account for the effects of multiple tests (n = 16), we consider *P* values smaller than an *α*-level of 0.0005 (0.01/20) as significant. Fold enrichment or depletion of TE insertions in putatively selected compartments was estimated by comparing the observed values to the median value of random outcomes.

Additionally, we tested whether the derived allele frequency (DAF) of TE insertions in putatively selected genomic compartments (exonic regions, CNEs) differed from control regions (intergenic spacers). The DAF for each insertion site was calculated by dividing the number of strains in which the insertion was present by the sample size of the data set (ngs_te_mapper: n = 166; TEMP: n = 53). Following previous efforts testing whether CNEs are cold spots of point mutation (Drake et al. 2006; Casillas et al. 2007), the null hypothesis of no difference in DAF between “selected” and “control” compartments was tested using a nonparametric Wilcoxon rank sum test. DAF tests of TE insertion allele frequencies in CNEs versus spacers were performed separately for intronic and intergenic regions. As in related work (Petrov et al. 2011; Kofler et al. 2012; Cridland et al. 2013), we assumed all TE insertions represent the derived state since, with the exception of the *INE-1* family that is not studied here (Singh et al. 2005; Wang et al. 2007), few TE insertions in *D. melanogaster* are thought to have occurred prior to speciation (Caspi and Pachter 2005; Bergman and Bensasson 2007; Sackton et al. 2010). Rare TE insertions spanning intron/exon on intergenic/exon boundaries were excluded from DAF analysis because of their low sample sizes. However, TE insertions spanning CNE/spacer boundaries were relatively common, and thus were analyzed as distinct class and compared with TEs contained fully within spacers.

All graphical and statistical analyses were performed in the R programming environment (version 3.4.0) (https://www.r-project.org/; last accessed June 6, 2018).

## Results

### TE Insertions Are Depleted in Conserved Noncoding Elements

To understand whether selective constraints on noncoding DNA influence patterns of TE insertion, we analyzed the abundance of nonreference TEs insertions in different functional genomic compartments of the *D. melanogaster* genome. We first assigned nonreference TE insertions in normally recombining regions to functional compartments based on gene and conserved element annotations (see Materials and Methods for details). We then tested for depletion of nonreference TE insertions in genomic regions with putatively higher levels of functional constraint (i.e., exonic regions, CNEs) by comparing observed numbers of TEs in these regions to an empirical null distribution based of 10,000 random permutations of the observed TE insertion data sets. Finally, we tested whether the DAF spectrum for TE insertions in genomic regions with putatively higher levels of functional constraint was skewed toward rarer alleles, as would be expected if TE insertions in these regions were weakly negatively selected.

Recent studies have shown that no single bioinformatic system can comprehensively identify all nonreference TE insertions in resequencing data (Nelson et al. 2017; Rishishwar et al. 2017). Therefore, we used two independent nonreference TE insertion data sets in our analysis, ngs_te_mapper (Linheiro and Bergman 2012) and TEMP (Zhuang et al. 2014), both derived from the same sample of strain-specific genome sequences isolated from a North American population of *D. melanogaster* (Mackay et al. 2012). Unlike related data sets for the DGRP population that do not map TE insertion breakpoints to exact locations (Cridland et al. 2013; Rahman et al. 2015), the ngs_te_mapper and TEMP data sets analyzed here both use TE-flanking region junction information contained in split reads to annotate TE insertions with highest possible resolution (the TSD; see Bergman 2012 for discussion). The high positional accuracy of the ngs_te_mapper and TEMP data sets improves identification of allelic insertions occupying the same insertion site in different strains and assignment of TE insertion sites to specific genomic compartments. We did not filter either data set to remove regions with identity-by-descent to another strain or residual heterozygosity within strain because these issues affect only ∼10% of sites in the DGRP genomes (Lack et al. 2015), are expected to influence our abundance and DAF analyses only by small factors, and can only bias our results if these regions are nonrandomly associated with functional compartments. The ngs_te_mapper and TEMP data sets used here are largely nonoverlapping, with only 869 insertion sites in common (14.3–18.7% of each data set). Because of the largely nonoverlapping nature of these data sets, together with biases associated with merging data sets and the inability to interpret merged data sets in the context of previous benchmarking results, we analyzed both data sets independently to address how robust our results are to TE detection methods. The numbers of nonreference TE insertions, nucleotides, and proportion of the genome spanned are shown for exons, introns, and intergenic regions in table 1 and for CNEs and spacers in noncoding regions in table 2.

**Table 1:** TE Insertions in Normal Recombination Regions

Region	Coverage (bp)	% Normal Rec. Genome	# ngs_te_mapper TE	% ngs_te_mapper TE	# TEMP TE	% TEMP TE
Exon	27502613	26.4	399	6.6	278	6
Intron	38960671	37.4	2,743	45.3	2,153	46.3
Intron/exon	n.a.	n.a.	5	0.1	7	0.2
Intergenic	37804929	36.3	2,905	47.9	2,210	47.5
Intergenic/exon	n.a.	n.a.	9	0.1	4	0.1
Total	104268213	100	6,061	100	4,652	100

Note.—Columns contain the coverage (in bp) and percent of the normally recombining genome covered for exonic, intronic, and intergenic regions followed by the number and percent of TE insertions found fully in exonic, intronic, and intergenic regions or spanning intron/exon and intergenic/exon boundaries for both ngs_te_mapper and TEMP. Overlap categories have “n.a.” for coverage and percent of the normally recombining genome covered since boundaries between compartments do not occupy any space. Regions of the reference genome identified by RepeatMasker as TE were subtracted from all compartments and any nonreference TE in these regions were excluded from all analyses. Regions of normal recombination were defined by Cridland et al. (2013).

**Table 2:** TE Insertions in Noncoding Regions with Normal Recombination

Region	Coverage (bp)	% Normal Rec. Noncoding Genome	# ngs_te_mapper TE	% ngs_te_mapper TE	# TEMP TE	% TEMP TE
Intronic CNE	14093340	18.4	747	13.2	500	11.5
Intronic spacer	24867331	32.4	1,842	32.6	1,458	33.4
Intronic CNE/spacer	n.a.	n.a.	154	2.7	195	4.5
Intergenic CNE	14749396	19.2	813	14.4	577	13.2
Intergenic spacer	23055533	30	1,928	34.1	1,447	33.2
Intergenic CNE/spacer	n.a.	n.a.	164	2.9	186	4.3
Total	76765600	100	5,648	100	4,363	100

Note.—Columns contain the coverage (in bp) and percent of the normally recombining noncoding genome covered by CNEs and spacers for introns and intergenic regions followed by the number and percent of TE insertions found fully in CNEs and spacers or spanning CNE/spacer boundaries for both ngs_te_mapper and TEMP. Overlap categories have “n.a.” for coverage and percent of the normally recombining noncoding genome covered since boundaries between compartments do not occupy any space. Regions of the reference genome identified by RepeatMasker as TE and any nonreference TE in these regions were excluded from all compartments. Regions of normal recombination were defined by Cridland et al. (2013).

**Fig. 1.**
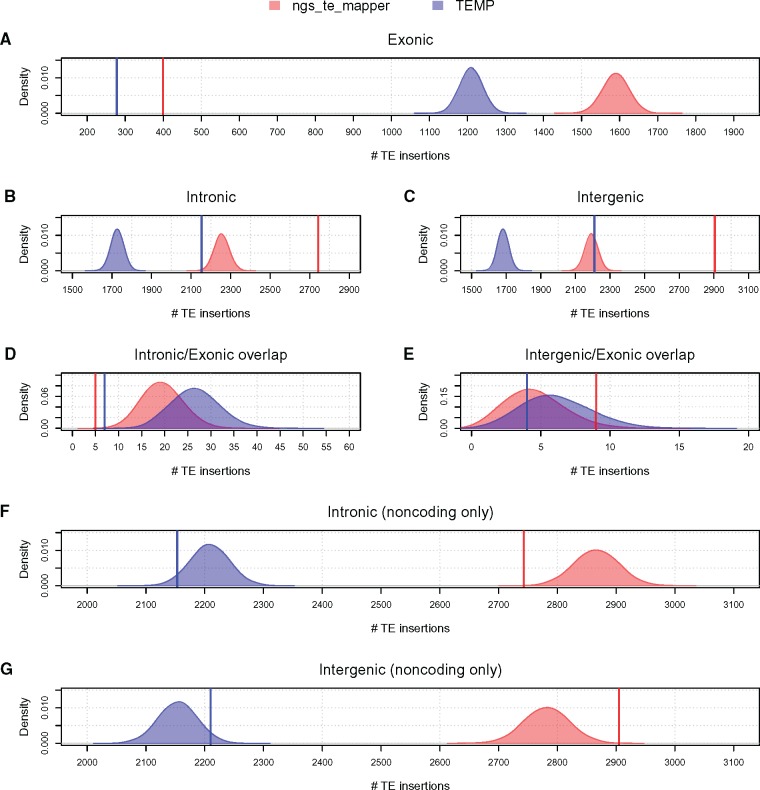
—TEs in normally recombining regions of the *Drosophila melanogaster* genome are depleted in exonic and intronic regions. Observed numbers of TEs in different genomic compartments are shown as vertical lines for ngs_te_mapper (red) and TEMP (blue). Empirical null distributions of the numbers of TEs in different genomic compartments in 10,000 random permutations are shown as density plots for ngs_te_mapper (red) and TEMP (blue). All permutation analyses were restricted to normally recombining regions of the *D. melanogaster* genome as defined by Cridland et al. (2013). Permutation analyses were conducted across all compartments (*A–E*), or in noncoding regions only (*F* and *G*). Observed and simulated numbers of TEs were counted in exonic regions (*A*), intronic regions (*B* and *F*), intergenic regions (*C* and *G*), intronic/exonic boundaries (*D*), and intergenic/exonic boundaries (*E*). Observed TEs overlapping intron/exon boundaries or intergenic/exon boundaries were excluded from permutation analyses in noncoding regions only (*F* and *G*). Regions of the reference genome identified by RepeatMasker as TE sequence and any nonreference TE in these regions were also excluded from all permutation analyses.

As a positive control, we first tested whether the previously reported depletion of TE insertions in *D. melanogaster* exonic regions (Lipatov et al. 2005; Kofler et al. 2012; Cridland et al. 2013) could be observed in the ngs_te_mapper and TEMP data sets using our permutation procedure. As shown in table 1, several hundred TE insertions can be found in exonic regions in natural populations of *D. melanogaster* (see also Kofler et al. 2012; Cridland et al. 2013). Nevertheless, we observed a clear depletion of TE insertions in exonic regions relative to random expectations (fig. 1*A*), coupled with a concomitant excess in intronic regions (fig. 1*B*) and intergenic regions (fig. 1*C*). We estimate a 4-fold (*P* < 1*e*-04) and 4.35-fold (*P* < 1*e*-04) reduction in TEs in exonic regions relative to the median of random outcomes for the ngs_te_mapper and TEMP data sets, respectively (fig. 1*A*). We also detected evidence for a significant depletion of TE insertions spanning intron/exon boundaries (fig. 1*D*) for both ngs_te_mapper (4.6-fold reduction, *P* = 1*e*-04) and TEMP (5.9-fold reduction, *P* < 1*e*-04), consistent with the presence of “hazardous zones” for TE insertion near intron–exon junctions shown previously in humans (Zhang et al. 2011). In contrast, we observed no significant depletion of TEs at intergenic/exon boundaries (fig. 1*E*; ngs_te_mapper: *P* = 0.98; TEMP: *P* = 0.27). These results support previous analyses that TEs are selectively eliminated from exonic regions (Lipatov et al. 2005; Petrov et al. 2011; Kofler et al. 2012; Cridland et al. 2013), and demonstrate that our approach can detect selective constraints on TE insertions that are assumed to exist in the *D. melanogaster* genome.

We next investigated whether our data provide evidence that purifying selection eliminates a higher proportion of TEs in intronic regions relative to intergenic regions, by permuting the locations of TEs in noncoding regions only. We observed a trend toward fewer TE insertions in intronic regions relative to random expectation (fig. 1*F*) with a corresponding excess in intergenic regions (fig. 1*G*) in both data sets. The magnitude of this effect was weak but significant in the ngs_te_mapper data set (1.05-fold reduction, *P* = 3*e*-04), and of a similar magnitude but not significant in the TEMP data set (1.02-fold reduction, *P* = 0.05). Our results support those of Kofler et al. (2012) who similarly observed a weak but significant reduction in numbers of TE insertions in intronic regions relative to intergenic regions using pool-seq data, but differ from Cridland et al. (2013) who observed more TEs in intronic regions relative to intergenic regions using strain-specific genome data. Together, these results suggest that the TE density in *D. melanogaster* intronic regions is weakly reduced relative to random expectations, but that the proportion of TEs eliminated from intronic regions is not sufficiently large for the effect to be reliably identified in all population genomic data sets.

**Fig. 2.**
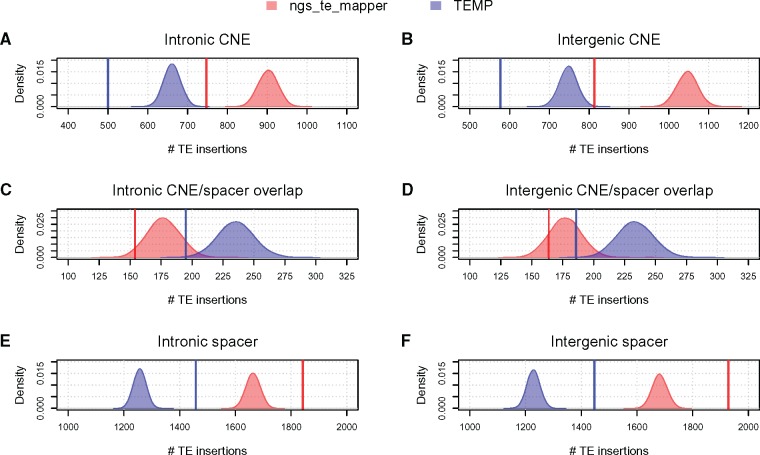
—TEs in normally recombining regions of the *Drosophila melanogaster* genome are depleted in conserved noncoding elements. Observed numbers of TEs in different noncoding compartments are shown as vertical lines for ngs_te_mapper (red) and TEMP (blue). Empirical null distributions of the numbers of TEs in different noncoding compartments in 10,000 random permutations are shown as density plots for ngs_te_mapper (red) and TEMP (blue). All permutation analyses were restricted to normally recombining regions of the *D. melanogaster* genome as defined by Cridland et al. (2013). Permutation analyses were conducted across intronic regions only (*A*, *C*, and *E*) or intergenic regions only (*B*, *D*, and *F*). Observed and simulated numbers of TEs were counted in CNEs (*A* and *B*), CNE/spacer boundaries (*C* and *D*), or spacers (*E* and *F*). The TEMP data set has higher number of observed and expected CNE/spacer overlaps (*C* and *D*) despite having fewer TE insertions overall because of a larger average TSD length (7.71 bp) relative to ngs_te_mapper (4.73 bp). Observed TEs overlapping intron/exon boundaries or intergenic/exon boundaries were excluded from these analyses. Regions of the reference genome identified by RepeatMasker as TE sequence and any nonreference TE in these regions were also excluded from all permutation analyses.

Finally, we tested whether TE insertions were depleted in CNEs relative to spacer regions (fig. 2). For this analysis, we permuted TE insertions separately within intronic regions and within intergenic regions and accounted for TE insertions spanning CNE/spacer boundaries. We identified several hundred TE insertions that exist in CNEs in both intronic and intergenic regions (table 2). Nonetheless, we found evidence for a significant depletion in the density of TEs in CNEs in both intronic regions (fig. 2*A*; ngs_te_mapper: 1.21-fold reduction, *P* < 1*e*-04; TEMP: 1.31-fold reduction, *P* < 1*e*-04) and intergenic regions (fig. 2*B*; ngs_te_mapper: 1.3-fold reduction, *P* < 1*e*-04; TEMP: 1.3-fold reduction, *P* < 1*e*-04). We also observed a weak but nonsignificant trend for fewer TE insertions overlapping CNE/spacer boundaries relative to random expectation in intronic regions (fig. 2*C*; ngs_te_mapper: 1.18-fold reduction, *P* = 0.04; TEMP: 1.23-fold reduction, *P* = 0.002). Fewer TE insertions overlapping CNE/spacer boundaries relative to expectations were also observed in intergenic regions, with data for TEMP but not ngs_te_mapper showing a significant effect (fig. 2*D*; ngs_te_mapper: 1.16-fold reduction, *P* = 0.16; TEMP: 1.28-fold reduction, *P* = 1*e*-04). Correspondingly, we also observe that TE insertions in both data sets are overrepresented in spacers in both intronic regions (fig. 2*E*; ngs_te_mapper: 1.11-fold excess; TEMP: 1.15-fold excess) and intergenic regions (fig. 2*F*; ngs_te_mapper: 1.83-fold excess; TEMP: 1.17-fold excess). Overall, these results suggest that while some CNEs tolerate disruption by large TE insertions, constraints on CNEs are substantial enough to eliminate enough TE insertions in CNEs to bias the distribution of observed TE insertions toward spacers in noncoding regions of the *D. melanogaster* genome.

### Allele Frequencies of TE Insertions are Similar across Different Functional Compartments of the *D. melanogaster* Genome

Additional evidence for purifying selection acting to shape the landscape of TE insertions can potentially be obtained from investigating the allele frequencies of TE insertions in population samples. Population genetics theory predicts that natural selection will prevent new deleterious alleles from reaching high population frequency (Fay et al. 2001). If polymorphic TE insertions are weakly negatively selected, they should be skewed toward lower allele frequencies in regions under higher selective constraint such as exonic regions and CNEs relative to control regions that have weaker functional constraint. A skew in the frequency of *D. melanogaster* SNPs toward rarer alleles has previously been observed in CNEs relative to spacers (Casillas et al. 2007) and in replacement sites relative to silent sites (Huang et al. 2014). However, small indels showed no tendency to be skewed toward rarer alleles in CNEs relative to spacers (Casillas et al. 2007), suggesting a similar distribution of fitness effects for small indels in both types of noncoding region.

**Fig. 3.**
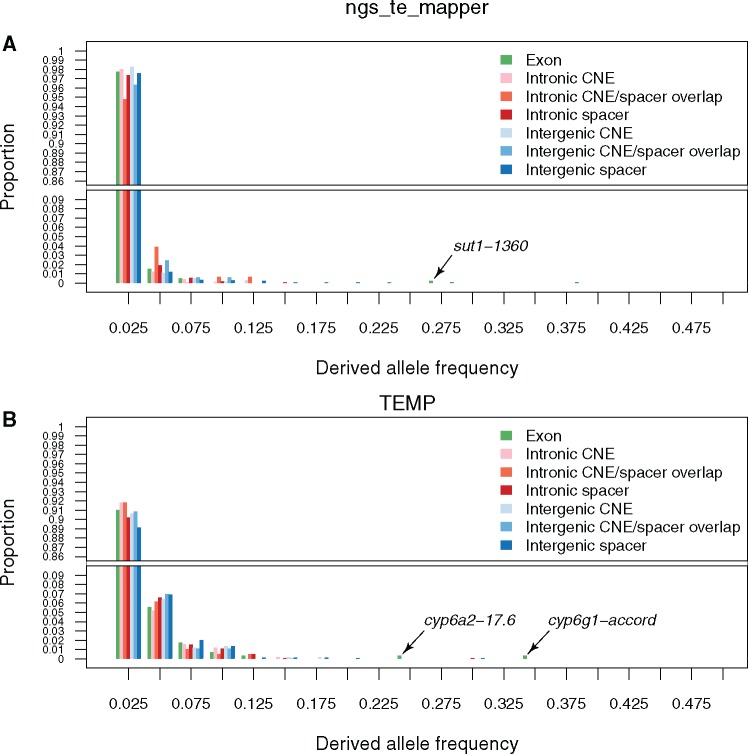
—The derived allele frequency (DAF) spectrum for TE insertions is similar across different compartments of the *Drosophila melanogaster* genome. DAF spectra are shown for TE insertions predicted by ngs_te_mapper (*A*) or TEMP (*B*). Allele frequency classes are shown on the *X* axis, and the proportion of TE insertions observed in a particular compartment of the genome at that allele frequency is shown on the *Y* axis. Note that the *Y* axis is split to allow better visualization of the proportion of higher allele frequency classes.

Figure 3 shows the DAF spectra for TE insertions in different functional compartments across the *D. melanogaster* genome. Consistent with classical restriction mapping and in situ hybridization studies (reviewed in Charlesworth and Langley 1989 and Nuzhdin 1999) and recent strain-specific population genomic data (Cridland et al. 2013), both data sets show the expected pattern for TE insertion alleles to be skewed toward rare alleles in all genomic compartments. However, clear differences are observed between ngs_te_mapper (fig. 3*A*) and TEMP (fig. 3*B*) in the overall shape of the DAF spectra across all compartments, with a skew toward more rare alleles in the ngs_te_mapper data set relative to TEMP. We interpret overall differences in DAF spectra between TE data sets to result primarily from the higher false negative rate for ngs_te_mapper relative to TEMP (Nelson et al. 2017) (see Discussion). Regardless of the cause(s) of systematic differences in the DAF spectra across methods, comparison of DAF spectra across genomic compartments *within* a data set should not be substantially compromised, since all compartments are affected by the similar methodological biases in TE detection.

We first assessed whether the expected skew toward lower allele frequencies could be observed for TE insertion in exonic regions. For this and all subsequent DAF spectra analyses, we used the frequency distribution of TE insertions in intergenic spacers as a control, based on abundance results above showing this compartment was under the weakest selective constraint for TE insertion. As shown in figure 3, we find no significant differences between the DAF spectra for TEs in exonic regions in either data set: (ngs_te_mapper: *W* = 391,158.5, *P* = 0.43; TEMP: *W* = 205,299.5, *P* = 0.36). One possibility for the lack of skew toward rarer alleles for TEs in exonic regions is the presence of a small number of unusually high-frequency exonic TE insertions that are potentially involved in adaptation to insecticide resistance (arrows, fig. 3*A* and *B*) (ngs_te_mapper: *1360* in *sut1*, Steele et al. 2015; TEMP: *17.6* in *cyp6a2*, Waters et al. 1992; Delpuech et al. 1993; Wan et al. 2014, *accord* in *cyp6g1*, Daborn et al. 2002; Chung et al. 2006). When these putatively adaptitive outlier loci are excluded, TEs in exonic regions still do not show a consistent skew toward rarer alleles relative to those in intergenic spacers regions: (ngs_te_mapper: *W* = 389,232.5, *P* = 0.5; TEMP: *W* = 203,853.5, *P* = 0.27). These results suggest that the distribution of fitness effects for exonic TE insertions that are not strongly deleterious does not differ substantially from those in intergenic spacers (see also Lipatov et al. 2005).

Next, we tested whether the DAF spectrum for TE insertions in CNEs differed from those in noncoding spacer regions. In this analysis, we also considered the DAF spectrum of TE insertions that spanned CNE/spacer boundaries because this overlap class is reasonably common and also exhibits a trend toward being depleted in TE insertions (see above). As shown in figure 3, we found no significant differences in the DAF spectra for TEs in CNEs relative to those in spacer intervals in both intronic regions (ngs_te_mapper: *W* = 671,827, *P* = 0.19; TEMP: *W* = 358,690, *P* = 0.29) and intergenic regions (ngs_te_mapper: *W* = 767,402.5, *P* = 0.2; TEMP: *W* = 411,058, *P* = 0.31). Likewise, the DAF spectra for TEs overlapping CNE/spacer boundaries did not differ from TEs fully contained in spacer intervals in both intronic regions (ngs_te_mapper: *W* = 141,937, *P* = 0.98; TEMP: *W* = 139,781.5, *P* = 0.46) and intergenic regions (ngs_te_mapper: *W* = 157,028.5, *P* = 0.83; TEMP: *W* = 132,093, *P* = 0.44). Similar to previous results for small indels (Casillas et al. 2007), these results imply that the distribution of fitness effects on TE insertions wholly or partially contained in CNEs is not substantially different from that operating on spacer regions in noncoding DNA.

## Discussion

Here, we show that the abundance of TE insertions is significantly reduced relative to random expectation in two distinct genomic compartments with known or suspected function: exonic regions and CNEs. In contrast, we find no clear signature for a skew toward lower allele frequencies for TEs in these genomic compartments when compared with regions of the genome under the lowest level of selective constraint. Our results are consistent either with 1) nonrandom transposition causing TEs to avoid functional compartments like exonic regions and CNEs, or 2) a mode of purifying selection that differentially eliminates TE insertions from functional regions but leaves behind polymorphic TEs insertions that have a similar distribution of fitness effects across genomic compartments.

Support for purifying selection driving the patterns we observe comes from the facts that the majority of spontaneous mutations in *D. melanogaster* genes are caused by TEs (Ashburner et al. 2005) (proving that transposition can occur in functional regions), and that TE insertions are skewed toward lower allele frequencies relative to SNPs from the same population (Aquadro et al. 1986, 1992; Langley and Aquadro 1987; Langley et al. 1988; Schaeffer et al. 1988; Cridland et al. 2013). Moreover, TEs in *D. melanogaster* only show weak target site preferences for short AT-rich motifs (Linheiro and Bergman 2012), which argues against the nonrandom transposition model. The only *D. melanogaster* TE family known to have strong nonrandom insertion biases—the *P* element (Spradling et al. 1995; Bellen et al. 2004; Kofler et al. 2015)—was excluded from our analysis for this reason. Additionally, recent analysis of de novo TE insertion in *D. melanogaster* mutation accumulation lines found no association between transposition rate and exon content, and only one TE family (*copia*) showed an association with chromatin state (Adrion et al. 2017). Adrion et al. (2017) did find a marginally significant negative association between transposition rate and GC-content at the 10-kb scale. Coupled with the weak AT-bias of TE target site motifs and the fact that exons and CNEs are more GC-rich than their flanking regions (Casillas et al. 2007; Zhu et al. 2009), it is possible that base composition may contribute to the patterns of TE depletion seen in these functional compartments. However, the magnitude of differences in GC-content in the high-recombination regions studied here between noncoding regions (GC: 0.40) and exons (GC: 0.49) or between spacers (GC: 0.39) and CNEs (GC: 0.42) does not appear sufficient to explain the >14.1-fold increase in TE abundance in noncoding regions relative to exons or the >2.3-fold increase in TE abundance in spacers relative to CNEs. On balance, we conclude that purifying selection is the more likely explanation for the depletion of TEs observed in exons and CNEs. If this interpretation is correct, our results provide the first systematic evidence that selective constraints on CNEs influence the landscape of TE insertion in a eukaryote genome, and provide new evidence supporting the conclusion that CNEs are functionally constrained and not mutational cold spots.

Our conclusions are derived from two largely nonoverlapping TE insertion data sets (ngs_te_mapper and TEMP), indicating they are not dependent on the idiosyncrasies of a single method for calling TE insertions in short-read resequencing data. Nevertheless, it is important to consider how our results may be affected by the imperfect state of the art in TE calling in terms of positional accuracy and false negative rates (Nelson et al. 2017; Rishishwar et al. 2017). It is unlikely that the depletion of TE insertions we observe is due to imprecise annotation of the TE insertions analyzed here, since underrepresentation of TEs in exonic regions has been observed previously using a variety of different classical and genomic approaches (Aquadro et al. 1986, 1992; Langley and Aquadro 1987; Langley et al. 1988; Schaeffer et al. 1988; Bartolome et al. 2002; Kaminker et al. 2002; Lipatov et al. 2005; Kofler et al. 2012; Cridland et al. 2013; Zhuang et al. 2014). Likewise, if false negative rates are constant across genomic compartments, false negatives are unlikely to generate the abundance patterns we observe. For this to be the case, the allele frequency of TE insertions would need to be skewed toward lower frequencies in compartments with higher levels of constraint, so that a higher relative proportion of singleton TE insertion sites would fail to be detected in compartments under higher constraint (leading to an artifactually lower number of insertion sites in high constraint regions). We find no evidence for a skew toward lower DAF in compartments with higher levels of constraint in our data (fig. 3). False negative rates may, however, vary across functional compartments, for example, if higher SNP density in regions under lower constraint reduces read mapping quality and increase false negative rates. This potential bias cannot explain our results since it would lead to an enrichment of TE insertions in regions with high constraint, which is the opposite of the pattern observed here.

Although we observe the expected pattern of depletion of TE insertions in regions with higher constraint, we find no difference in the DAF spectra between highly constrained and weakly constrained compartments within either the ngs_te_mapper or TEMP data sets. As above, it is unlikely that positional inaccuracy or false negatives can explain the lack of difference in the DAF spectra between exonic regions or CNEs and spacers. The high positional accuracy of the ngs_te_mapper and TEMP data sets mitigates against mis-assignment of TEs to the wrong compartment, which could in principle cause the DAF spectra for different compartments to appear more similar than they really are. Furthermore, in the case of CNEs, we accounted for potential blurring of compartment assignment by showing that the DAF spectra of TEs spanning CNE/spacer boundaries have similar allele frequencies to TEs fully contained within CNEs. Additionally, while it is clear that false negatives distort the DAF spectrum toward rare alleles (Emerson et al. 2008), if the false negative rate is uniform across the genome, false negatives should affect the DAF spectra for all functional compartments in a similar way. It is formally possible that one reason we failed to detect a real skew toward lower DAF in more highly constrained regions is because SNP-induced reduction in mapping quality increases false negative TE detection rates in regions with lower constraint, although we are unaware of any evidence supporting this possibility. It is also possible that our analysis lacks power to detect a real skew toward rare alleles in the DAF for TE insertions in exons and CNEs. Previous results studying TE insertions in *D. melanogaster* exons using pool-seq data showed a reduction in median allele frequencies relative to those found in intergenic regions (Kofler et al. 2012), however exonic TE insertions studied using pool-PCR suggested their allele frequencies did not differ substantially from nonexonic TE insertions with similar genomic properties (Lipatov et al. 2005). Future studies may reveal whether these discrepancies are related to differences in methodology or truly reflect similarity in TE insertion allele frequencies across compartments. If clear differences can be identified in the frequency of TE insertions in exons and CNEs relative to intergenic spacers, it would be interesting to estimate the strength of purifying selection acting on TE insertions in these compartments (Keightley and Eyre-Walker 2007).

Importantly, we observed systematic differences in the DAF spectrum across different nonreference TE insertion data sets, which has not been discussed sufficiently as an issue in population genomic analysis of TE evolution. Specifically, we find that the DAF for ngs_te_mapper is skewed more toward lower frequencies that the DAF for TEMP (fig. 3*A* vs *B*). We do not interpret this difference among data sets to result from lower positional accuracy of ngs_te_mapper relative to TEMP artificially splitting alleles from the same insertion site into several different insertion sites each at lower allele frequency, since both data sets use split-read information. Rather it is more likely this difference in DAF among data sets results from the higher false negative rate for ngs_te_mapper (58% on simulated data; Nelson et al. 2017) relative to TEMP (10% on simulated data; Nelson et al. 2017). This observation cautions against naive use of allele frequency data from short-read TE insertion detection methods to test predictions of population genetic models, since the precise shape of the frequency spectrum may be determined by false negative rates of TE detection methods rather than any particular evolutionary force (Emerson et al. 2008). This result also motivates more advanced methods to estimate the TE frequency spectra that incorporate false negative detection rates, similar to methods for estimating the frequency spectrum of SNPs that incorporate false positive rates due to sequencing error (Kim et al. 2011; Nielsen et al. 2012).

Our twin findings of depletion of TEs in functional elements like exonic regions and CNEs coupled with a lack of a skew toward rarer alleles in these regions suggests that the selective mechanism controlling location of TEs in the *D. melanogaster* genome may be decoupled from the forces governing allele frequencies of polymorphic alleles (Petrov et al. 2011). Among competing theories for selective forces acting on TE insertions (Nuzhdin 1999; Lee and Langley 2010), it is easiest to interpret the depletion of TEs in exonic regions as being due to the direct effects of TE insertion (Petrov et al. 2011; Kofler et al. 2012) and the same logic should hold for depletion of TEs in CNEs. However, the similarity of DAF spectra in different genomic compartments is consistent with the remainder of TE insertions that are not eliminated from functional elements being governed by a number of evolutionary mechanisms. Polymorphic TE insertions could be at similar allele frequencies in different compartments simply because they inserted at similar distributions of times in the past (Bergman and Bensasson 2007; Kofler et al. 2012; Blumenstiel et al. 2014). Alternatively, the similar DAF spectra of polymorphic TE insertions in different genomic compartments could reflect similar distributions of selective effects that are independent of the precise location of a TE insertion, which might be expected if the deleterious effects of TE insertion are caused by ectopic exchange events (Petrov et al. 2011; Kofler et al. 2012) or local epigenetic silencing spreading from TE insertions (Lee 2015; Lee and Karpen 2017). While our work does not resolve these widely debated alternatives, it does reveal that the selective effects of TE insertion on conserved elements in noncoding DNA should be factored into future models explaining TE evolution in *D. melanogaster* and other species. 

## Supplementary Material

Supplementary data are available at *Genome Biology and Evolution* online.

## Supplementary Material

Supplementary Data

## References

[evy104-B1] Adrion JR, Song MJ, Schrider DR, Hahn MW, Schaack S. 2017 Genome-wide estimates of transposable element insertion and deletion rates in *Drosophila melanogaster*. Genome Biol Evol. 9(5):1329–1340. 28338986 10.1093/gbe/evx050 PMC5447328

[evy104-B2] Aquadro CF, Desse SF, Bland MM, Langley CH, Laurie-Ahlberg CC. 1986 Molecular population genetics of the alcohol dehydrogenase gene region of *Drosophila melanogaster*. Genetics 114:1165–1190. 3026893 10.1093/genetics/114.4.1165 PMC1203034

[evy104-B3] Aquadro CF, Jennings RM, Bland MM, Laurie CC, Langley CH. 1992 Patterns of naturally occurring restriction map variation, dopa decarboxylase activity variation and linkage disequilibrium in the Ddc gene region of *Drosophila melanogaster*. Genetics 132:443–452. 1358752 10.1093/genetics/132.2.443 PMC1205148

[evy104-B4] Arnold CD, et al 2013 Genome-wide quantitative enhancer activity maps identified by STARR-seq. Science 339(6123):1074–1077. 23328393 10.1126/science.1232542

[evy104-B5] Ashburner M, Golic KG, Hawley RS. 2005 Drosophila: a laboratory handbook. Cold Spring Harbor (NY): Cold Spring Harbor Laboratory Press.

[evy104-B6] Barron MG, Fiston-Lavier A-S, Petrov DA, Gonzalez J. 2014 Population genomics of transposable elements in Drosophila. Annu Rev Genet. 48:561–581. 25292358 10.1146/annurev-genet-120213-092359

[evy104-B7] Bartolome C, Maside X, Charlesworth B. 2002 On the abundance and distribution of transposable elements in the genome of *Drosophila melanogaster*. Mol Biol Evol. 19(6):926–937. 12032249 10.1093/oxfordjournals.molbev.a004150

[evy104-B8] Bellen HJ, et al 2004 The BDGP gene disruption project: single transposon insertions associated with 40% of Drosophila genes. Genetics 167(2):761–781. 15238527 10.1534/genetics.104.026427 PMC1470905

[evy104-B9] Bergman CM. 2012 A proposal for the reference-based annotation of de novo transposable element insertions. Mob Genet Elem. 2(1):51–54. 10.4161/mge.19479 PMC3383450 22754753

[evy104-B10] Bergman CM, Bensasson D. 2007 Recent LTR retrotransposon insertion contrasts with waves of non-LTR insertion since speciation in *Drosophila melanogaster*. Proc Natl Acad Sci U S A. 104(27):11340–11345. 17592135 10.1073/pnas.0702552104 PMC2040900

[evy104-B11] Bergman CM, Kreitman M. 2001 Analysis of conserved noncoding DNA in Drosophila reveals similar constraints in intergenic and intronic sequences. Genome Res. 11(8):1335–1345. 11483574 10.1101/gr.178701

[evy104-B12] Bergman CM, et al 2002 Assessing the impact of comparative genomic sequence data on the functional annotation of the Drosophila genome. Genome Biol. 3(12):research0086.1. 12537575 10.1186/gb-2002-3-12-research0086 PMC151188

[evy104-B13] Bergman CM, Quesneville H, Anxolabehere D, Ashburner M. 2006 Recurrent insertion and duplication generate networks of transposable element sequences in the *Drosophila melanogaster* genome. Genome Biol. 7:R112. 17134480 10.1186/gb-2006-7-11-r112 PMC1794594

[evy104-B14] Blumenstiel JP, Chen X, He M, Bergman CM. 2014 An age-of-allele test of neutrality for transposable element insertions. Genetics 196(2):523–538. 24336751 10.1534/genetics.113.158147 PMC3914624

[evy104-B15] Brody T, et al 2012 Use of a Drosophila genome-wide conserved sequence database to identify functionally related cis-regulatory enhancers. Dev Dyn. 241(1):169–189. 22174086 10.1002/dvdy.22728 PMC3243966

[evy104-B16] Casillas S, Barbadilla A, Bergman CM. 2007 Purifying selection maintains highly conserved noncoding sequences in Drosophila. Mol Biol Evol. 24(10):2222–2234. 17646256 10.1093/molbev/msm150

[evy104-B17] Caspi A, Pachter L. 2005 Identification of transposable elements using multiple alignments of related genomes. Genome Res. 16(2):260–270. 16354754 10.1101/gr.4361206 PMC1361722

[evy104-B18] Charlesworth B, Langley CH. 1989 The population genetics of Drosophila transposable elements. Annu Rev Genet. 23:251–287. 2559652 10.1146/annurev.ge.23.120189.001343

[evy104-B19] Chung H, et al 2006 Cis-regulatory elements in the Accord retrotransposon result in tissue-specific expression of the Drosophila melanogaster insecticide resistance gene Cyp6g1. Genetics 175(3):1071–1077. 17179088 10.1534/genetics.106.066597 PMC1840086

[evy104-B20] Cridland JM, Macdonald SJ, Long AD, Thornton KR. 2013 Abundance and distribution of transposable elements in two Drosophila QTL mapping resources. Mol Biol Evol. 30(10):2311–2327. 23883524 10.1093/molbev/mst129 PMC3773372

[evy104-B21] Cridland JM, Thornton KR, Long AD. 2015 Gene expression variation in *Drosophila melanogaster* due to rare transposable element insertion alleles of large effect. Genetics 199(1):85–93. 25335504 10.1534/genetics.114.170837 PMC4286695

[evy104-B22] Daborn PJ, et al 2002 A single p450 allele associated with insecticide resistance in Drosophila. Science 297(5590):2253–2256. 12351787 10.1126/science.1074170

[evy104-B23] Delpuech JM, Aquadro CF, Roush RT. 1993 Noninvolvement of the long terminal repeat of transposable element 17.6 in insecticide resistance in Drosophila. Proc Natl Acad Sci U S A. 90(12):5643–5647. 8390673 10.1073/pnas.90.12.5643 PMC46777

[evy104-B24] Drake JA, et al 2006 Conserved noncoding sequences are selectively constrained and not mutation cold spots. Nat Genet. 38(2):223–227. 16380714 10.1038/ng1710

[evy104-B25] Elliott TA, Gregory TR. 2015 What’s in a genome? The C-value enigma and the evolution of eukaryotic genome content. Philos Trans R Soc B 370(1678):20140331. 10.1098/rstb.2014.0331 PMC4571570 26323762

[evy104-B26] Emberly E, Rajewsky N, Siggia ED. 2003 Conservation of regulatory elements between two species of Drosophila. BMC Bioinformatics 4:57. 14629780 10.1186/1471-2105-4-57 PMC302112

[evy104-B27] Emerson JJ, Cardoso-Moreira M, Borevitz JO, Long M. 2008 Natural selection shapes genome-wide patterns of copy-number polymorphism in *Drosophila melanogaster*. Science 320(5883):1629–1631. 18535209 10.1126/science.1158078

[evy104-B28] Fay JC, Wyckoff GJ, Wu CI. 2001 Positive and negative selection on the human genome. Genetics 158(3):1227–1234. 11454770 10.1093/genetics/158.3.1227 PMC1461725

[evy104-B29] Geyer P, Green M, Corces V. 1990 Tissue-specific transcriptional enhancers may act in trans on the gene located in the homologous chromosome: the molecular basis of transvection in Drosophila. EMBO J. 9:2247–2256. 2162766 10.1002/j.1460-2075.1990.tb07395.x PMC551949

[evy104-B30] Gramates LS et al 2017 FlyBase at 25: looking to the future. Nucleic Acids Res. 45(D1):D663–D671. 27799470 10.1093/nar/gkw1016 PMC5210523

[evy104-B31] Haddrill PR, Halligan DL, Tomaras D, Charlesworth B. 2007 Reduced efficacy of selection in regions of the Drosophila genome that lack crossing over. Genome Biol. 8(2):R18. 17284312 10.1186/gb-2007-8-2-r18 PMC1852418

[evy104-B32] Huang W, et al 2014 Natural variation in genome architecture among 205 *Drosophila melanogaster* Genetic Reference Panel lines. Genome Res. 24(7):1193–1208. 24714809 10.1101/gr.171546.113 PMC4079974

[evy104-B33] Kaminker JS, et al 2002 The transposable elements of the *Drosophila melanogaster* euchromatin: a genomics perspective. Genome Biol. 3(12):research0084.1. 12537573 10.1186/gb-2002-3-12-research0084 PMC151186

[evy104-B34] Keightley PD, Eyre-Walker A. 2007 Joint inference of the distribution of fitness effects of deleterious mutations and population demography based on nucleotide polymorphism frequencies. Genetics 177(4):2251–2261. 18073430 10.1534/genetics.107.080663 PMC2219502

[evy104-B35] Kim SY, et al 2011 Estimation of allele frequency and association mapping using next-generation sequencing data. BMC Bioinformatics 12(1):231. 21663684 10.1186/1471-2105-12-231 PMC3212839

[evy104-B36] Kofler R, Betancourt AJ, Schlotterer C. 2012 Sequencing of pooled DNA samples (pool-seq) uncovers complex dynamics of transposable element insertions in *Drosophila melanogaster*. PLoS Genet. 8(1):e1002487. 22291611 10.1371/journal.pgen.1002487 PMC3266889

[evy104-B37] Kofler R, Hill T, Nolte V, Betancourt AJ, Schlötterer C. 2015 The recent invasion of natural *Drosophila simulans* populations by the P-element. Proc Natl Acad Sci U S A. 112(21):6659–6663. 25964349 10.1073/pnas.1500758112 PMC4450375

[evy104-B38] Kuhn RM, Haussler D, Kent WJ. 2013 The UCSC genome browser and associated tools. Brief Bioinform. 14(2):144–161. 22908213 10.1093/bib/bbs038 PMC3603215

[evy104-B39] Lack JB, et al 2015 The Drosophila genome nexus: a population genomic resource of 623 *Drosophila melanogaster* genomes, including 197 from a single ancestral range population. Genetics 199(4):1229–1241. 25631317 10.1534/genetics.115.174664 PMC4391556

[evy104-B40] Langley CH, Aquadro CF. 1987 Restriction-map variation in natural populations of *Drosophila melanogaster*: white-locus region. Mol Biol Evol. 4:651–663. 2895415 10.1093/oxfordjournals.molbev.a040467

[evy104-B41] Langley CH, et al 1988 Naturally occurring variation in the restriction map of the Amy region of *Drosophila melanogaster*. Genetics 119:619–629. 17246437 10.1093/genetics/119.3.619 PMC1203447

[evy104-B42] Lee YCG. 2015 The role of piRNA-mediated epigenetic silencing in the population dynamics of transposable elements in *Drosophila melanogaster*. PLoS Genet. 11(6):e1005269. 26042931 10.1371/journal.pgen.1005269 PMC4456100

[evy104-B43] Lee YCG, Karpen GH. 2017 Pervasive epigenetic effects of Drosophila euchromatic transposable elements impact their evolution. Elife 6:e25762. 10.7554/eLife.25762 PMC5505702 28695823

[evy104-B44] Lee YCG, Langley CH. 2010 Transposable elements in natural populations of *Drosophila melanogaster*. Philos Trans R Soc Lond B Biol Sci. 365(1544):1219–1228. 20308097 10.1098/rstb.2009.0318 PMC2871824

[evy104-B45] Lerman DN, Feder ME. 2005 Naturally occurring transposable elements disrupt hsp70 promoter function in *Drosophila melanogaster*. Mol Biol Evol. 22(3):776–783. 15574805 10.1093/molbev/msi063

[evy104-B47] Linheiro RS, Bergman CM. 2012 Whole genome resequencing reveals natural target site preferences of transposable elements in *Drosophila melanogaster*. PLoS One 7(2):e30008. 22347367 10.1371/journal.pone.0030008 PMC3276498

[evy104-B48] Lipatov M, Lenkov K, Petrov DA, Bergman CM. 2005 Paucity of chimeric gene-transposable element transcripts in the *Drosophila melanogaster* genome. BMC Biol. 3:24. 16283942 10.1186/1741-7007-3-24 PMC1308810

[evy104-B49] Mackay TFC, et al 2012 The *Drosophila melanogaster* genetic reference panel. Nature 482(7384):173–178. 22318601 10.1038/nature10811 PMC3683990

[evy104-B50] Makunin IV, Shloma VV, Stephen SJ, Pheasant M, Belyakin SN. 2013 Comparison of ultra-conserved elements in Drosophilids and vertebrates. PLoS One 8(12):e82362. 24349264 10.1371/journal.pone.0082362 PMC3862641

[evy104-B51] Negre N, et al 2011 A cis-regulatory map of the Drosophila genome. Nature 471(7339):527–531. 21430782 10.1038/nature09990 PMC3179250

[evy104-B52] Nelson MG, Linheiro RS, Bergman CM. 2017 McClintock: an integrated pipeline for detecting transposable element insertions in whole-genome shotgun sequencing data. G3 (Bethesda) 7:2749–2762. 28637810 10.1534/g3.117.043893 PMC5555480

[evy104-B53] Nielsen R, Korneliussen T, Albrechtsen A, Li Y, Wang J. 2012 SNP calling, genotype calling, and sample allele frequency estimation from new-generation sequencing data. PLoS One 7(7):e37558. 22911679 10.1371/journal.pone.0037558 PMC3404070

[evy104-B54] Nuzhdin SV. 1999 Sure facts, speculations, and open questions about the evolution of transposable element copy number. Genetica 107(1–3):129. 10952206

[evy104-B55] Petrov DA, Fiston-Lavier A-S, Lipatov M, Lenkov K, Gonzalez J. 2011 Population genomics of transposable elements in *Drosophila melanogaster*. Mol Biol Evol. 28(5):1633–1644. 21172826 10.1093/molbev/msq337 PMC3080135

[evy104-B56] Presgraves DC. 2005 Recombination enhances protein adaptation in *Drosophila melanogaster*. Curr Biol. 15(18):1651–1656. 16169487 10.1016/j.cub.2005.07.065

[evy104-B57] Quinlan AR, Hall IM. 2010 BEDTools: a flexible suite of utilities for comparing genomic features. Bioinformatics 26(6):841–842. 20110278 10.1093/bioinformatics/btq033 PMC2832824

[evy104-B59] Rahman R, et al 2015 Unique transposon landscapes are pervasive across *Drosophila melanogaster* genomes. Nucleic Acids Res. 43(22):10655–10672. 26578579 10.1093/nar/gkv1193 PMC4678822

[evy104-B60] Rishishwar L, Mario-Ramrez L, Jordan IK. 2017 Benchmarking computational tools for polymorphic transposable element detection. Brief Bioinformatics 18:908–918. 27524380 10.1093/bib/bbw072 PMC5808724

[evy104-B61] Sackton TB, et al 2010 Population genomic inferences from sparse high-throughput sequencing of two populations of *Drosophila melanogaster*. Genome Biol Evol. 1(0):449–465. 10.1093/gbe/evp048 PMC2839279 20333214

[evy104-B62] Schaeffer SW, Aquadro CF, Langley CH. 1988 Restriction-map variation in the Notch region of *Drosophila melanogaster*. Mol Biol Evol. 5(1):30–40. 2833676 10.1093/oxfordjournals.molbev.a040475

[evy104-B63] Siepel A, et al 2005 Evolutionarily conserved elements in vertebrate, insect, worm, and yeast genomes. Genome Res. 15(8):1034–1050. 16024819 10.1101/gr.3715005 PMC1182216

[evy104-B64] Singh ND, Arndt PF, Petrov DA. 2005 Genomic heterogeneity of background substitutional patterns in *Drosophila melanogaster*. Genetics 169(2):709–722. 15520267 10.1534/genetics.104.032250 PMC1449091

[evy104-B66] Spradling AC, et al 1995 Gene disruptions using P transposable elements: an integral component of the Drosophila genome project. Proc Natl Acad Sci U S A. 92(24):10824–10830. 7479892 10.1073/pnas.92.24.10824 PMC40524

[evy104-B67] Steele LD, et al 2015 Selective sweep analysis in the genomes of the 91-R and 91-C *Drosophila melanogaster* strains reveals few of the usual suspects in dichlorodiphenyltrichloroethane (DDT) resistance. PLoS One 10(3):e0123066. 25826265 10.1371/journal.pone.0123066 PMC4380341

[evy104-B68] Tyner C, et al 2017 The UCSC Genome Browser database: 2017 update. Nucleic Acids Res. 45:D626–D634. 27899642 10.1093/nar/gkw1134 PMC5210591

[evy104-B69] Wan H, et al 2014 Nrf2/Maf-binding-site-containing functional Cyp6a2 allele is associated with DDT resistance in *Drosophila melanogaster*. Pest Manag Sci. 70(7):1048–1058. 24038867 10.1002/ps.3645

[evy104-B70] Wang J, Keightley PD, Halligan DL. 2007 Effect of divergence time and recombination rate on molecular evolution of Drosophila INE-1 transposable elements and other candidates for neutrally evolving sites. J Mol Evol. 65(6):627. 17896069 10.1007/s00239-007-9028-6

[evy104-B71] Waters LC, Zelhof AC, Shaw BJ, Ch’ang LY. 1992 Possible involvement of the long terminal repeat of transposable element 17.6 in regulating expression of an insecticide resistance-associated P450 gene in Drosophila. Proc Natl Acad Sci U S A. 89(11):4855–4859. 1317576 10.1073/pnas.89.11.4855 PMC49186

[evy104-B72] Zhang Y, Romanish MT, Mager DL. 2011 Distributions of transposable elements reveal hazardous zones in mammalian introns. PLoS Comput Biol. 7(5):e1002046. 21573203 10.1371/journal.pcbi.1002046 PMC3088655

[evy104-B73] Zhu L, et al 2009 Patterns of exon-intron architecture variation of genes in eukaryotic genomes. BMC Genomics 10:47. 19166620 10.1186/1471-2164-10-47 PMC2636830

[evy104-B74] Zhuang J, Wang J, Theurkauf W, Weng Z. 2014 TEMP: a computational method for analyzing transposable element polymorphism in populations. Nucleic Acids Res. 42(11):6826–6838. 24753423 10.1093/nar/gku323 PMC4066757

